# Editorial

**Published:** 1868-06-15

**Authors:** 


### *EDITORIAL.*

Correspondents will accept our sincere thanks for their favors, but must excuse a little delay in their publication. Each accepted will appear at the earliest possible period.

The unexpected length of Dr. Bosworth's article compels us to reserve the remainder until next number.

Dr. Baxter's case of Tetanus, treated by *Calabar Bean*, will attract general attention. We believe, at its date, it is the fourth case reported. The reporter, Dr. Curran, does not state, what we understand to be the fact, that the use of the *Bean* was suggested by Prof. Gunn. The Editorial impression is that the result is balanced between the *Morphine* and the *Bean*. This is *Nota Bene*.

The Apostle thinks the excessive amount of labor expended by the Faculty of the Reform School is such that it will not do to *elevate* the fees. A combination is anticipated with the Vinegar Factory.

